# Care coordination and communication for Native American patients with cancer

**DOI:** 10.1093/oncolo/oyaf407

**Published:** 2026-01-25

**Authors:** Mariah P Daley, Sheryl Buckner, Katie Keyser, Vanessa Wright, Katy Fisher-Cunningham, Amber Anderson-Buettner, Mark P Doescher, Amanda Janitz, Stephnie Dartez, Shondra McCage, Tara Matthews, Kelly Irwin, Dorothy A Rhoades, Ryan D Nipp

**Affiliations:** Department of Medicine, Division of Hematology & Oncology, University of Oklahoma (OU) Health Campus, OU Health Stephenson Cancer Center, Oklahoma City, OK 73104, United States; Department of Nursing Academic Programs, Fran and Earl Ziegler College of Nursing, University of Oklahoma Health Campus, Oklahoma City, OK 73117, United States; Department of Medicine, Division of Hematology & Oncology, University of Oklahoma (OU) Health Campus, OU Health Stephenson Cancer Center, Oklahoma City, OK 73104, United States; Department of Nursing Academic Programs, Fran and Earl Ziegler College of Nursing, University of Oklahoma Health Campus, Oklahoma City, OK 73117, United States; Department of Nursing Academic Programs, Fran and Earl Ziegler College of Nursing, University of Oklahoma Health Campus, Oklahoma City, OK 73117, United States; Biostatistics & Epidemiology, University of Oklahoma Health Campus, Oklahoma City, OK 73104, United States; Department Family and Preventive Medicine, University of Oklahoma (OU) Health Campus, OU Health Stephenson Cancer Center, Oklahoma City, OK 73104, United States; Department of Biostatistics and Epidemiology, Hudson College of Public Health, University of Oklahoma Health Campus, Oklahoma City, OK 73104, United States; American Indian Navigation, OU Health, OU Health Stephenson Cancer Center, Oklahoma City, OK 73104, United States; Division of Public Health, The Chickasaw Nation Department of Health, Ada, OK 74820, United States; Medisaw Case Management, The Chickasaw Nation Medical Center, Ada, OK 74820, United States; Department of Psychiatry, Massachusetts General Hospital and Harvard Medical School, Boston, MA 02115, United States; Department of Medicine, University of Oklahoma Health Campus, Native American Center for Cancer Health Excellence, OU Health Stephenson Cancer Center, Oklahoma City, OK 73104, United States; Department of Medicine, Division of Hematology & Oncology, University of Oklahoma (OU) Health Campus, OU Health Stephenson Cancer Center, Oklahoma City, OK 73104, United States

**Keywords:** Native American, cancer, supportive care, patient navigation, cancer outcomes research

## Introduction

Native American populations, including American Indian and Alaska Native individuals, face significant disparities in cancer outcomes.^1^ From 2016 to 2020, the American Indian and Alaska Native population experienced the highest overall cancer incidence and cancer mortality rates in the United States,^2–6^ with cancer representing the leading cause of death for those aged 55-74.^7^ In Oklahoma, the state with the second largest proportion of U.S. residents identifying as American Indian, the cancer mortality rate is estimated at 1.5 times higher than the United States all-races rate.^6^^,^^8^^,^^9^ We use the terms “American Indian” and “Alaska Native” (AI/AN) throughout this commentary in accordance with the federal Office of Management and Budget definitions of race and ethnicity.^10^ Other descriptors, such as “Native American” or “First American,” are also used to refer to the descendants of the pre-colonial inhabitants of the United States. Few Alaska Native persons reside in Oklahoma, and thus, we chose to use the term “American Indian” when referring specifically to population within the state.

The American Indian and Alaska Native population faces significant challenges in cancer care.^10^ Notably, 33% of Oklahoma American Indian individuals reside in rural areas,^11^^,^^12^ higher than the national average of 14%.^11^^,^^12^ Related to this higher rural distribution, patients often experience fragmented care, limited access to treatment, and lack of supportive services.^13–16^ Clinician shortages, limited oncology facilities, and transportation challenges further compound these disparities.^17^ Cancer care coordination may help alleviate these challenges but has rarely been studied in the health systems serving American Indian patients.^18^ Therefore, an urgent need exists to address cancer care coordination challenges for these patients, highlighting the necessity to develop and test innovative solutions tailored to the specific needs and cultural context of this population.

## Challenges experienced by American Indian and Alaska Native patients with cancer

Despite overall improvements in life expectancy, American Indian and Alaska Native populations have persistently ­experienced shorter lifespans and higher cancer mortality.^1^ Studies suggest that American Indian and Alaska Native ­individuals are more likely to receive a late-stage diagnosis of screening-detectable cancers, particularly colorectal and prostate cancers.^1^^,^^19–23^ Additionally, research has shown that American Indian and Alaska Native patients with cancer are less likely to receive guideline-concordant cancer care.^24–27^ Many American Indian and Alaska Native individuals live in rural, economically disadvantaged, or medically underserved areas, with reduced access to oncology care.^29^^,^^30^ These barriers place patients at risk for significant financial hardship, further contributing to morbidity and mortality.^28–30^ Addressing these interconnected issues requires a multifaceted, coordinated approach to improve cancer outcomes.^28^^,^^31^^,^^32^

Many American Indian and Alaska Native patients receive healthcare directly from federally administered Indian Health Service (IHS) programs, tribally administered facilities and programs that receive IHS funding, or Urban Indian Organizations, which are independent nonprofit community-based clinics that contract with the IHS to serve urban populations.^12^^,^^14^^,^^15^ Collectively, these are known as the ITU system. ITU facilities serve nearly 2.6 million American Indian and Alaska Native individuals across 37 states. However, most lack the resources to provide comprehensive oncology services,^12^ resulting in referrals to non-ITU facilities through the Purchased/Referred Care program.^33^ The involvement of multiple clinicians across systems can cause care delays and fragmentation.^13^^,^^34^ In Oklahoma, which has 39 federally recognized tribes, coordination of care between cancer centers and the complex ITU system is critically important.

The University of Oklahoma (OU) Health Stephenson Cancer Center is home to the state’s only cancer navigation program specifically for American Indian patients.^9^ This program facilitates oncology referrals from the ITUs and has previously addressed financial hardship in this population.^18^ Although the navigation program expedites referrals for tests and clinical visits, significant gaps persist in two-way communication between the cancer center and referring ITU teams. Consequently, strategies are needed to enhance care coordination and communication to fit this unique context.

## Innovative care coordination and communication approaches for providing specialty-level care

Collaborative care models are structured, team-based management strategies that integrate communication, coordination, and shared decision-making across disciplines to improve patient outcomes.^35^ These models have improved outcomes in mental health, heart failure, and diabetes by enhancing communication and integrating supportive services.^36^^,^^37^ Although underutilized in oncology, these models hold promise for complex patient populations.^38^^,^^39^ In cancer care, research suggests that collaborative models can improve access to treatment and integrate psychosocial support.^40^^,^^41^ Their success in managing multifaceted conditions underscores the potential to adapt these approaches to cancer care, especially for populations navigating intricate referral networks.^35^ Such models emphasize team-based collaboration, facilitating integrated specialty-level care and greater alignment across clinicians by promoting patient-centered care.^42–44^ By improving access to specialists and enhancing communication among clinicians, care coordination and communication models offer an innovative and promising solution for enhancing the care of American Indian persons with cancer.

## Developing a care coordination and communication program for American Indian patients with cancer

At the Stephenson Cancer Center, the Native American Navigation Program was created with the support of two tribal nations to assist patients referred from ITU facilities. Navigators help with referrals, appointments, collecting records, and connecting patients with services. Despite these efforts, persistent challenges in care coordination and two-way communication remain across clinical systems.

Building on this foundation, our team previously pilot-implemented regular team meetings, or “huddles,” between the Stephenson Cancer Center and an ITU program. The coordination occurs through structured huddles involving navigators, care coordinators, and ITU representatives, streamlining communication without adding additional staff. Implementation challenges have included clarifying the meeting structure and purpose, securing encrypted patient data, resolving technical issues, and fostering cross-system collaboration.^45^

Expanding on these efforts, we are developing the Care Coordination and Communication Program in Oncology (C3PO) for Tribal Health Systems (NIH grant: U19MD020537).^45^ This program broadens interdisciplinary strategies to streamline care, foster rapid specialty access, and strengthen partnerships with ITU providers. The augmented communication within C3PO aims to build rapport and deepen understanding of patients’ needs and beliefs, thereby enhancing treatment decision-making and improving long-term outcomes. The program also tracks preliminary indicators, such as referral completion rates, time to first oncology visit, and patient navigation touchpoints. The huddles will occur separately for the six ITU programs that provide the most referrals, representing approximately 225 patients from more than 20 tribes annually, many from rural locations.

## Care Coordination and Communication Program in Oncology

**Figure 1.**
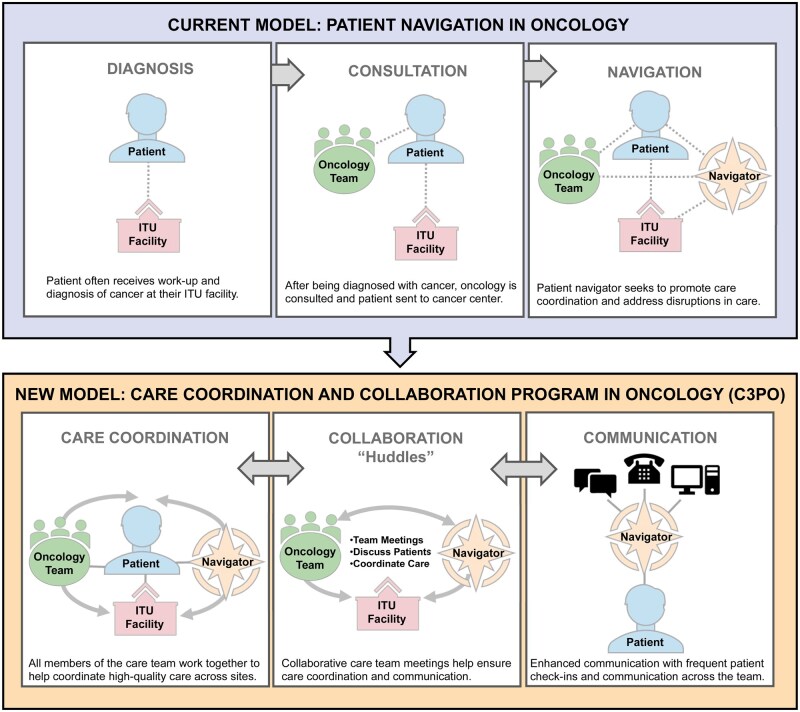
Model for Care Coordination and Communication Program in Oncology (C3PO). The model in the top box illustrates the current model of patient navigation in oncology. The patient may receive work-up and diagnosis of cancer at their ITU facility. Once the patient’s ITU has submitted a referral to the cancer center, the patient navigator at the cancer center seeks to promote care coordination and address disruptions in care. The model in the lower box highlights the new model of Care Coordination and Communication Program in Oncology (C3PO). This new model contains the steps in the top model, but also provide innovative additions in which the cancer center team works collaboratively with the ITU and navigation team to enhance care coordination and communication across sites. The new model incorporates collaborative care team meetings (also called “huddles”) to help ensure care coordination and communication. Finally, in this new model, the patient navigator helps improve cross-communication with frequent patient check-ins and communication across the teams.

We designed C3PO (Figure 1) to transform cancer care for American Indian patients by fostering communication among patients, navigators, care coordinators, oncology teams, and ITU facilities. In C3PO, care coordinators meet with patients biweekly to discuss symptoms, social needs (e.g., finances, transportation, housing), and cancer care challenges, while monthly C3PO huddles will connect oncology and ITU teams to bridge care gaps and foster communication across sites.

In these C3PO huddles, ITU and Stephenson Cancer Center staff review patient care plans, trajectories, and needs. Over time, we will develop and refine the meeting discussions, which center on patient concerns, health trajectory, and plans to ensure adequate follow-up. These efforts lay the foundation for structured interdisciplinary communication that prioritizes patient-centered care. We hypothesize that C3PO will enhance patient satisfaction, reduce missed oncology visits, shorten time to treatment initiation, and manage treatment interruptions. Consistent communication may also support culturally respectful decision-making and improved long-term outcomes.

## Potential barriers to implementing a care coordination and communication program

Implementing a care coordination and communication model for American Indian patients with cancer will likely encounter challenges necessitating careful attention. Historical mistrust rooted in unethical medical practices may lead to hesitancy, especially if Western medicine approaches overshadow traditional healing efforts.^46^^,^^47^ Through clearer communication and focused education to clarify huddle goals, we aim to enhance understanding and trust across the health system, while improving outcomes.

Other barriers include limited integration of traditional and Western medicine, a lack of clinicians skilled in American Indian culture, and economic and geographic challenges.^48^ To address these concerns, C3PO incorporates tribal staff with firsthand experience navigating ITU systems and prioritizes cultural respect, affordability, and clinician awareness. This expertise provides a unique perspective and understanding of the American Indian patient experience. By promoting integration and collaboration, C3PO represents a promising, culturally informed strategy for comprehensive and holistic cancer care in Oklahoma and beyond.

## Conclusion: a step toward transformative care for American Indian patients with cancer

Addressing the cancer disparities among American Indian populations requires a comprehensive, culturally responsive approach that integrates medical and social considerations. Care coordination and communication models offer a promising strategy to bridge gaps in access to specialty care while enhancing communication across health systems, cancer care teams, and patients. This model has the potential to improve the quality of care, foster trust, and strengthen patient engagement.^45^ Implementing innovative care coordination and communication approaches, such as C3PO, has the potential to transform care by systematically and collaboratively addressing the clinical needs of American Indian individuals with cancer. Challenges related to historical mistrust, economic and geographic barriers, and gaps in care will require continued attention. Fostering specialty-level care and ensuring enhanced communication are essential for advancing cancer care delivery, reducing disparities, and promoting improved oncologic outcomes for American Indian communities.

## Data Availability

*No new data were generated or analyzed in support of this research.*
